# Association between *PEG3* DNA methylation and high-grade cervical intraepithelial neoplasia

**DOI:** 10.1186/s13027-021-00382-3

**Published:** 2021-06-13

**Authors:** Claire Bosire, Adriana C. Vidal, Jennifer S. Smith, Dereje Jima, Zhiqing Huang, David Skaar, Fidel Valea, Rex Bentley, Margaret Gradison, Kimberly S. H. Yarnall, Anne Ford, Francine Overcash, Susan K. Murphy, Cathrine Hoyo

**Affiliations:** 1 Department of Health Behavior, Gillings School of Global Public Health, University of North Carolina, Chapel Hill, NC USA; 2 Department of Surgery, Cedars-Sinai Medical Center, Los Angeles, CA USA; 3 Department of Epidemiology, Gillings School of Global Public Health and Lineberger Comprehensive Cancer Center, University of North Carolina, Chapel Hill, NC USA; 4 Department of Biological Sciences, Center for Human Health and the Environment, North Carolina State University, Raleigh, NC USA; 5 Department of Obstetrics and Gynecology, Division of Reproductive Sciences, Duke University School of Medicine, Durham, NC USA; 6 Department of Obstetrics and Gynecology, Virginia Tech Carilion School of Medicine, Roanoke, VA USA; 7 Department of Pathology, Duke University School of Medicine, Durham, NC USA; 8 Department of Family Medicine and Community Health, Duke University School of Medicine, Durham, NC USA

**Keywords:** Cervical intraepithelial neoplasia, Gene methylation, Imprinted gene, Human papillomavirus

## Abstract

**Background:**

Epigenetic mechanisms are hypothesized to contribute substantially to the progression of cervical intraepithelial neoplasia (CIN) to cervical cancer, although empirical data are limited.

**Methods:**

Women (*n* = 419) were enrolled at colposcopic evaluation at Duke Medical Center in Durham, North Carolina. Human papillomavirus (HPV) was genotyped by HPV linear array and CIN grade was ascertained by biopsy pathologic review. DNA methylation was measured at differentially methylated regions (DMRs) regulating genomic imprinting of the *IGF2/H19*, *IGF2AS, MESTIT1/MEST, MEG3, PLAGL1/HYMAI, Kv*DMR and *PEG10, PEG3* imprinted domains, using Sequenom-EpiTYPER assays. Logistic regression models were used to evaluate the associations between HPV infection, DMR methylation and CIN risk overall and by race.

**Results:**

Of the 419 participants, 20 had CIN3+, 52 had CIN2, and 347 had ≤ CIN1 (CIN1 and negative histology). The median participant age was 28.6 (IQR:11.6) and 40% were African American. Overall, we found no statistically significant association between altered methylation in selected DMRs and CIN2+ compared to ≤CIN1. Similarly, there was no significant association between DMR methylation and CIN3+ compared to ≤CIN2. Restricting the outcome to CIN2+ cases that were HR-HPV positive and p16 staining positive, we found a significant association with *PEG3* DMR methylation (OR: 1.56 95% CI: 1.03–2.36).

**Conclusions:**

While the small number of high-grade CIN cases limit inferences, our findings suggest an association between altered DNA methylation at regulatory regions of *PEG3* and high grade CIN in high-risk HPV positive cases.

**Supplementary Information:**

The online version contains supplementary material available at 10.1186/s13027-021-00382-3.

## Background

Screening and treatment of cervical intraepithelial neoplasia (CIN) has significantly reduced invasive cervical cancer (ICC) incidence and mortality [1]. However, an estimated 13,800 new ICC cases and 4290 deaths were expected in the United States in 2020 [2]. ICC continues to disproportionally affect Hispanic and Black women as compared to White and Asian-American women [3].

Current cervical cancer screening guidelines include cytology-based screening, with the addition of high-risk human papillomavirus (HPV) testing in women 30 years and older to increase sensitivity for the detection of high grade CIN (CIN2+) or cancer [4]. Cytology-based tests have relatively low single-use sensitivity for detecting CIN2+ which could delay appropriate treatment [5, 6]. HPV testing is more sensitive than cytology in detecting CIN2+ but has relatively lower specificity for CIN2+ [7], detecting transient infections which could lead to unnecessary referrals to colposcopy. Besides a strategy to triage HPV positive women, molecular markers that can improve the prediction of progression of CIN to ICC are needed. Further, to increase the participation of under-screened populations, screening strategies that include vaginal self-sampling with high-risk HPV (HR-HPV) testing are a viable alternative [8]. It is important to identify molecular markers to triage HPV positive women using self-sampling devices in the absence of cervical cytology.

Methylation biomarkers are promising tools for the detection of CIN2+ [9, 10]. DNA methylation plays an important role in the regulation of gene expression and cancer development [11]. In high-grade precancer and cancer, imprinted differentially methylated regions (DMRs) are often deregulated, showing gains or losses in methylation that affect changes in expression and sometimes alter imprint status [12]. DNA methylation changes can result in either silencing of the only active allele or, aberrantly activating expression of the normally silenced allele, which may double the gene dosage [13].

DNA methylation in genes and HPV testing have been investigated as triage methods to improve risk stratification of HR-HPV positive women. Studies summarizing the performance of DNA methylation in CIN2 and CIN3 detection have reported significantly higher DNA methylation in CIN2+ and CIN3+ compared to ≤ CIN1 [10, 14]. We previously reported that aberrant methylation at DMRs that regulate the expression of imprinted genes including *Insulin-like Growth Factor 2* (which expresses IGF2, a potent mitogen), *Paternally Expressed Gene 1/Mesoderm-Specific Transcript* (*PEG1/MEST*) and *Paternally Expressed Gene 3* (*PEG3*) were independent predictors of CIN2/3 and ICC in a cross-sectional study of Tanzanian women with CIN and ICC [15, 16]. We also reported a negative association between CIN1 regression and methylation at *Insulin-Like Growth Factor 2, Antisense (IGF2AS)* and *Paternally Expressed Gene 10* (*PEG10)* DMR in a study of CIN1 cases drawn from the Duke University Cervical Intraepithelial Neoplasia Cohort Study (CINCS) [17]. Preliminary analyses have found dysregulated expression of imprinted genes involved in tumor suppression (e.g., *Pleiomorphic Adenoma Gene-Like* 1 (*PLAGL1)* and *Hydatidiform Mole Associated and Imprinted* (*HYMA*I)) in cervical cancer specimen compared to normal cervical tissue [18], and *Maternally Expressed Gene 3 (MEG3)* hypermethylation has been implicated as a potential biomarker in cervical cancer [19]. While studies in *voltage-gated potassium channels* (*Kv) DMR* are limited, changes in methylation at *Kv* DMR have been positively associated with breast [20] and colorectal cancer [21].

Here, we examine whether aberrant DNA methylation at DMRs regulating genomic imprinting of *IGF2/H19, IGF2AS, MESTIT1/MEST, MEG3, PLAGL1/HYMAI, Kv DMR* and *PEG10, PEG3*, are associated with CIN (CIN2+ vs ≤ CIN1 and CIN3+ vs ≤ CIN2) in women participating in the multiethnic CINCS [22] overall, and in race-stratified analyses.

## Methods

### Study participants

Study participants were recruited from all 10 Duke University and Duke colposcopy clinics in Durham County, North Carolina, from 2010 to 2012, as previously described [22]. Briefly, all clinics used the same study protocol and the Duke University Pathology Laboratory for cytologic and histologic evaluation. To be eligible, study participants were initially screened for cervical abnormalities with the Thin-Prep liquid-based cytology test (Cytyc®). Inclusion criteria were: a visit to one of 10 colposcopy clinics following an abnormal Pap test of at least low grade squamous intraepithelial lesion (LSIL), age 18 years or older, and English or Spanish speaking. Questionnaires were written in English and a Spanish-speaking coordinator assisted and interpreted the questionnaire content to Spanish-speaking study participants. The self- and interviewer-administered instruments were identical in content. Women who did not intend to receive follow-up care in one of the 10 colposcopy clinics or moved out of the area for other reasons were excluded. Of the 1657 women with cytological abnormalities approached in the colposcopy clinic, 1303 were enrolled, a response rate of 79%. This study was approved by Duke University School of Medicine Institutional Review Board and informed consent was obtained from each study participant prior to enrollment in the study.

### Data collection

A standardized questionnaire that was either self- or interviewer-administered solicited information on risk factors such as: age, race, parity, yearly income, cigarette smoking, contraceptive use, dietary and sexual habits. Age was treated as a continuous variable; race/ethnicity was categorized as non-Hispanic Black; non-Hispanic White, Hispanic and other; parity was nulliparous or parous; HPV-DNA status was none, high-risk or low-risk HPV; and current smoking, contraceptive use and previous HPV vaccination were yes or no categories.

### Specimens

At the enrollment visit, exfoliation and collection of cervical cells was performed using a spatula and cytobrush. Cervical exfoliated cells were suspended in a vial containing ThinPrep® solution (Hologic®, Malborough, MA, USA) for cytological assessment. Colposcopy-directed biopsies were also obtained from the lesions. All specimens were tested for adequacy using the 2012 ASCCP guidelines [23]. The specimens were stored at 4 °C prior to HPV testing.

### Ascertainment of CIN and HPV genotyping

The biopsies underwent pathologic review to ascertain the presence of CIN. The hematoxylin and eosin-stained slides of individuals with cytological abnormalities were read by the study pathologist’s (RB) laboratory.

Testing for 37 HPV DNA subtypes was done at Johns Hopkins University, as previously described [22]. Following DNA extraction, HPV status was determined by targeted amplification of a 450 bp region of the HPV L1 genome using PGMY09/PGMY11 primers. Amplification of the human β-globin gene was included as an internal control for sample sufficiency. HPV genotyping was performed using HPV Linear Array (Roche Diagnostics, Branchburg, NJ, USA) [24, 25]; HPV genotypes 16, 18, 31, 33, 35, 39, 45, 51, 52, 56, 58, 59, 66, and 68 were considered high-risk or oncogenic genotypes, whereas HPV 6, 11, 26, 40, 42, 53, 54, 55, 61, 62, 64, 67, 69, 70, 72, 73, 81, 82, 83 and 84 were considered low-risk (LR) HPV types [26, 27].

### DMR DNA methylation

DNA methylation was measured at differentially methylated regions (DMRs) regulating genomic imprinting of *IGF2/H19*, *IGF2AS, MESTIT1/MEST, Kv* DMR, MEG3, PLAGL1/HYMAI and *PEG3* and PEG10 imprinted domains using Sequenom (San Diego, CA) EpiTYPER assays with the primers shown in Additional file 1. For each sample, 800 ng of DNA isolated from the exfoliated cervical cells were bisulfite converted using the EZ-96 DNA methylation kit (Zymo Research Corporation, Irvine, CA) to convert unmethylated DNA cytosine bases to uracil bases, leaving methylated cytosines unchanged as per manufacturer’s protocol. The bisulfite converted DNA was eluted into 40ul of elution buffer and transferred into 384-well plates. PCR was carried out using 20 ng bisulfite-converted DNA in a 10ul reaction volume with HotStarTaq DNA Polymerase (Qiagen; Valencia, CA). The PCR products were then treated with shrimp alkaline phosphatase (SAP, Sequenom, San Diego, CA) followed by transcription and T cleavage reactions according to the protocol from Sequenom. The cleanup and sequencing were performed according to the EpiTYPER user guide (Sequenom). The average methylation percentage from the CpG sites included in each DMR was used for analysis.

### Statistical analyses

Analyses were limited to 419 participants who had DNA methylation data, confirmed CIN status and covariate data including race/ethnicity and age. The primary contrast was CIN2+ vs ≤ CIN1. We also evaluated CIN3+ vs ≤ CIN2. Chi-squared and Fisher’s exact tests were used to compare socio-demographic characteristics of women with ≤CIN1 (CIN1 or no evidence of CIN) to those with CIN2, and CIN3+. HPV infections were grouped according to potential oncogenicity [26, 27] using the Bethesda criteria [28], as previously described [22]. Samples that failed assays owing to suboptimal DNA quality were excluded from analysis. A total of eight imprinted DMRs were considered a priori with five CpG sites for the *IGF2/H19* DMR; 10 CpGs for the *IGF2AS* DMR; 31 CpGs for the *MESTIT1/MEST* DMR; 27 CpG sites for the *Kv* DMR; 31 CpG sites for the *MEG3* DMR; eight CpG sites for the *PLAGL1/HYMAI* DMR; 11 CpG sites for the *PEG10* DMR; and 12 CpG sites for the *PEG3* DMR. We calculated summary methylation percentages across each candidate region. Mann-Whitney test was used to compare methylation differences across groups and receiver operating characteristic (ROC) curve analyses were used to evaluate how well methylation identified women with CIN2+ or CIN3 + .

We used logistic regression models to estimate odds ratios (OR) and corresponding 95% confidence intervals (CI) for the association between CIN (CIN2+ vs ≤ CIN1) and changes in DMR methylation. We also evaluated the association between methylation changes and CIN3+ vs ≤ CIN2. All models included methylation in 10% increments with adjustments made for race/ethnicity, age, parity, current smoking and HPV infection. In stratified analysis, we computed the OR and 95% CI for CIN2+ and CIN3+ and changes in DMR methylation separately for white and black women, adjusting for age, parity, current smoking and HPV infection. In additional analysis, we evaluated the association in CIN2+ cases that were p16 positive and HR-HPV positive.

We also estimated regression coefficients for the association between DMR methylation and HPV infection using mixed methods to allow for unconstrained model entry of individual CpGs at each DMR, with no HPV infection serving as the referent category. Statistical analyses were conducted using SAS 9.4 (SAS Institute, Cary, NC).

## Results

### Study participants

These results are based on the 419 participants who had DNA methylation data, confirmed CIN status and covariate data. There were seven beta-globin negative samples which were excluded from the analysis. Fourteen HR-HPV types were detected in 309 (74%) of participants, as single or multiple infections. Overall, the five most commonly detected HR-HPV types were HPV16 (55/419, 13%) and HPV66 (*n* = 55, 13%), HPV52 (*n* = 48, 11.5%), HPV51 (*n* = 46, 11%), and HPV39 (*n* = 44, 10.5%). The distribution of other HR-HPV was: HPV59 (*n* = 32, 7.6%), HPV31 (*n* = 31, 7.4%), HPV56 (*n* = 29, 6.9%), HPV58 (*n* = 28, 6.7%), HPV18 (*n* = 22, 5.3%), HPV35 (*n* = 18, 4.3%), HPV45 (*n* = 18, 4.3%), HPV68 (*n* = 17, 4.1%), and HPV33 (*n* = 12, 2.9%). Participants with measured HPV genotypes and DMR methylation data were comparable to those of the entire cohort with respect to age, HPV infection, yearly income, marital status and cigarette smoking (all *p* > 0.05).

The median age was 28.6 years (IQR 11.6) and did not differ significantly across the three CIN groups. Overall, 82.8% (*n* = 347) of women had ≤ CIN1, 12.4% (*n* = 52) had CIN2 and 4.8% (*n* = 20) had CIN3+ (Table 1). High-risk HPV infection prevalence was 70.9% in women with ≤ CIN1, 84.6% in CIN2 and 95% in CIN3 (*p* = .07). Women with CIN2+ were more likely to be parous than those with ≤CIN1 (*p* = .003), and to be current smokers (*p* < 0001). There were no significant differences by ethnicity or contraceptive use across the categories (*p* > .05).

**Table 1:** Characteristics of 419 study participants by cervical intraepithelial neoplasia (CIN) status

Characteristic	≤CIN1 (*n* = 347) n (%)	CIN2 (*n* = 52) n (%)	CIN3+ (*n* = 20) n (%)	*p* value
**Age** (median, IQR)	28.5 (11.5)	27.6 (11.6)	31.5 (15.1)	.23
**HPV infection**				.07
None	34 (9.8)	3 (5.8)	0 (0.0)	
High-risk	246 (70.9)	44 (84.6)	19 (95.0)	
Low-risk	67 (19.3)	5 (9.6)	1 (5.0)	
**Ethnicity**				.59
Non-Hispanic Black	145 (41.8)	18 (34.6)	5 (25.0)	
Non-Hispanic White	166 (47.8)	27 (51.9)	13 (65.0)	
Hispanic	16 (4.6)	4 (7.7)	1 (5.0)	
Other^a^	20 (5.8)	3 (5.8)	1 (5.0)	
**Current cigarette smoking**^b^				<.0001
Yes	46 (13.3)	19 (36.5)	7 (35.0)	
No	301 (86.7)	33 (63.5)	13 (65.0)	
**Parity**^b^				.003
Nulliparous	186 (53.6)	18 (35.3)	6 (30.0)	
Parous	161 (46.4)	33 (64.7)	14 (70.0)	
**Hormonal contraceptive use**^b^				.26
Yes	260 (79.8)	35 (77.8)	13 (72.2)	
No	66 (20.2)	10 (22.2)	5 (27.8)	

*Abbreviations*: *CIN* Cervical intraepithelial neoplasia, *IQR* Interquartile range, *HPV* Human papillomavirus. High risk HPV - 16, 18, 31, 33, 35, 39, 45, 51, 52, 56, 58, 59, 66, and 68

Low risk HPV - 6, 11, 26, 40, 42, 53, 54, 55, 61, 62, 64, 69, 70, 72, 73, 81, 82, 83 and 84

^a^Other includes Asian/Pacific Islanders and Native American

^b^Numbers do not add up to the total due to missing values

### Association between CpG methylation and CIN

Median methylation levels in CIN3+ vs  ≤ CIN2 and CIN2+ vs ≤ CIN1 varied across DMRs (Additional file 2). The ROC curve analysis of DMR methylation identification of CIN2+ and CIN3+ are shown in Additional file 3. The ROC area under the curve (AUC) values for CIN2+ were low for all DMRs, ranging from 0.54 (95%CI: 0.46–0.62) in *IGF2AS* to 0.50 (95%CI: 0.42–0.57) in *MESTITI/MEST*. The ROC AUC values for CIN3+ were also low, ranging from 0.62 (95%CI: 0.51–0.73) in *MEG3* to 0.51(95%CI: 0.38–0.64) in *PEG10*.

The multivariable-adjusted odds ratios and 95% CI for the associations between DMR methylation and CIN status, adjusted for age, HPV infection, race, smoking and parity are shown in Table 2. Comparing CIN2+ to ≤ CIN1 we found no statistically significant associations between DMR methylation and CIN2+. Comparing CIN3+ to ≤CIN2, we found no statistically significant associations with methylation changes. No significant associations were found when stratified by race. In additional analysis, we restricted the outcome to HR-HPV positive, p16 positive CIN2+ cases. In our study 47% of CIN2+ cases were stained for p16, and of these, 30/34 (88%) were positive. Comparing HR-HPV positive, p16+, CIN2+ cases to p16 negative/missing, ≤ CIN2 cases, we found a statistically significant association with *PEG3* methylation (OR: 1.56 95% CI: 1.03–2.36) (Additional file 4). No other significant associations were found.

**Table 2:** Odds ratios (OR) and 95% confidence intervals (CI) for associations between methylation levels of differentially methylated regions (DMR) regulating genomically imprinted genes and CIN

	All, ***N*** = 419 OR^a^ (95% CI)		Black, ***N*** = 168 OR^a^ (95% CI)		White, ***N*** = 206 OR^a^ (95% CI)	
Regulatory DMR	CIN2+ vs. ≤CIN1	CIN3+ vs. ≤CIN2	CIN2+ vs. ≤CIN1	CIN3+ vs. ≤CIN2	CIN2+ vs. ≤ CIN1	CIN3+ vs. ≤ CIN2
*PEG3*	1.16 (0.87–1.56)	0.79 (0.48–1.31)	1.31 (0.76–2.25)	1.71 (0.71–4.10)	1.13 (0.73–1.75)	0.70 (0.37–1.32)
*PLAGL1/HYMAI*	1.01 (0.73–1.38)	0.92 (0.51–1.66)	0.82 (0.44–1.52)	1.15 (0.51–2.58)	0.98 (0.63–1.52)	0.75 (0.35–1.61)
*Kv DMR*	1.21 (0.80–1.83)	0.91 (0.43–1.89)	1.22 (0.68–2.17)	1.47 (0.67–3.21)	1.58 (0.74–3.35)	0.88 (0.32–2.26)
*IGF2/H19*	1.15 (0.72–1.82)	0.76 (0.41–1.42)	0.43 (0.17–1.06)	0.43 (0.14–1.31)	1.61 (0.85–3.06)	0.93 (0.44–2.00)
*IGF2AS*	1.13 (0.93–1.38)	1.16 (0.82–1.65)	1.20 (0.85–1.71)	1.22 (0.73–2.03)	1.12 (0.85–1.48)	1.08 (0.72–1.61)
*MESTIT1/MEST*	1.08 (0.74–1.58)	0.90 (0.48–1.70)	1.14 (0.59–2.20)	0.36 (0.13–1.98)	0.88 (0.52–1.47)	1.11 (0.51–2.42)
*PEG10*	1.15 (0.73–1.81)	1.04 (0.48–2.23)	1.25 (0.50–3.13)	1.73 (0.45–6.74)	1.00 (0.54–1.85)	0.82 (0.32–2.08)
*MEG3*	1.01 (0.79–1.28)	1.38 (0.95–1.99)	0.95 (0.63–1.44)	1.06 (0.60–1.88)	1.13 (0.80–1.62)	1.58 (0.97–2.58)

*Abbreviations*: *CIN* Cervical intraepithelial neoplasia, *DMR* Differentially methylated region, *OR* Odds ratio

^a^OR adjusted for age, high risk HPV, race, smoking and parity

In stratified analyses, ORs for other races (Hispanic/Asian/Pacific Islanders/Native Americans) not shown (*n* = 45)

### Association between HPV infection and CpG methylation

High-risk HPV infections were significantly associated with altered methylation levels at *PLAGL1/HYMAI* DMR (β = − 2.25, SE = 0.92, *p* < .01) (Table 3). HR-HPV infections were also associated with *IGF2/H19* (β = 2.49, SE = 1.37, *p* = .07), *MESTIT1/MEST* (β = 1.46, SE = 0.83, *p* = .08) and *PEG10* (β = 0.84, SE = 0.5, *p* = .09*)* DMRs but these were not statistically significant*.* Among black women, the association between HR-HPV and methylation in DMRs was strongest for *PLAGL1/HYMAI* DMR (β = − 2.68, SE = 1.34, *p* = .04) and *Kv* DMR (β = 1.80, SE = 0.81, *p* = .03). Among white women, the strongest association between HR-HPV infection and methylation was at *MEG3* DMR (β = 3.82, SE = 1.04, *p* < .01).

**Table 3:** Regression coefficients and standard errors (SE) for the association between high risk and low risk HPV infections and DMR methylation

Regulatory DMR	All, *N* = 419 Coefficients (SE)		Blacks, *N* = 168 Coefficients (SE)		Whites, *N* = 206 Coefficients (SE)	
	HR-HPV	LR-HPV	HR-HPV	LR-HPV	HR-HPV	LR HPV
*PEG3*	−0.30 (0.77), *p* = .69	1.44 (0.90), *p* = .11	0.04 (1.21), *p* = .97	2.51 (1.47), *p* = .09)	No convergence	−1.21 (1.29), *p* = .35
*PLAGL1/HYMAI*	**−2.85 (0.92),** ***p*** **< .01**	−1.55 (1.08), *p* = .15	−**2.68 (1.34),** ***p*** **= .04**	−1.50 (1.68), *p* = .37	−1.95 (1.39), *p* = .16	−0.58 (1.60), *p* = .71
*Kv DMR*	0.37 (0.50), *p* = .47	0.33 (0.59), *p* = .58	**1.80 (0.81),** ***p*** **= .03**	2.51 (0.96), *p* < .01	−0.87 (0.74), *p* = .24	−1.39 (0.86), *p* = .11
*IGF2/H19*	2.49 (1.37), *p* = .07	2.80 (1.58), *p* = .08	3.02 (2.27), *p* = .18	3.11 (2.73), *p* = .25	2.84 (1.92), *p* = .14	2.84 (2.18), *p* = .19
*IGF2AS*	1.91 (1.35), *p* = .16	1.91 (1.56), *p* = .22	1.27 (2.13), *p* = .55	0.82 (2.54), *p* = .75	1.86 (1.98), *p* = .34	2.49 (2.22), *p* = .26
*MESTIT1/MEST*	1.46 (0.83), *p* = .08	0.95 (0.96), *p* = .33	−0.94 (1.37), *p* = .49	−1.55 (1.60), *p* = .33	2.23 (1.20), *p* = .06	1.68 (1.39), *p* = .23
*PEG10*	0.84 (0.50), *p* = .09	2.00 (0.59), *p* < .01	0.83 (0.78), *p* = .28	1.35 (0.94), *p* = .14	0.72 (0.75), *p* = .33	2.05 (0.85), *p* = .01
*MEG3*	−0.17 (1.17), *p* = .88	−2.08 (1.41) *p* = .14	−0.17 (1.17), *p* = .89	−2.08 (1.41), *p* = .14	**3.82 (1.04),** ***p*** **< .01**	3.25 (1.17), *p* < .01

*Abbreviations*: *DMR* Differentially methylated regions. *HR-HPV* High-risk human papillomavirus, *LR-HPV* Low-risk human papillomavirus, *SE* Standard error

Mixed models allowed for adjustment of individual CpGs at each DMR

No HPV infection served as the referent category

## Discussion

In this multiethnic cohort of women, we found a statistically significant association between altered methylation at *PEG3* DMR and CIN2+ in HR-HPV positive cases. No other statistically significant associations were found.

Studies have shown that CIN2+ and CIN3+ have higher levels of DNA methylation than CIN1 [10, 14]. A study summarizing the performance of DNA methylation of human genes and HPV virus in detecting CIN2+ and CIN3+ found that the DNA markers had high specificity to detect CIN lesions that were likely to progress [10].

We previously reported on the link between higher methylation of the *PEG3* DMR and ICC in Tanzanian women [15]. Our results further support the hypothesis that aberrant methylation of *PEG3* DMR may be an important factor in the CIN progression in HR-HPV positive women, with 10% increment in methylation at *PEG3* DMR associated with 1.56 times the odds of CIN2+. Evidence suggests that *PEG3* plays an important biological role in P53/c-myc mediated apoptosis, implicating *PEG3* functions as a tumor suppressor in carcinogenesis [29, 30].

The odds ratio for the association between methylation in *IGF2/H19* DMR and HR-HPV positive CIN2+ was 1.62 (95%CI: 0.82–3.20). This was not statistically significant. In the Tanzania study, we reported a significant association between methylation in IGF2/H19 imprinted domain and risk of CIN (OR: 1.51 95%CI:1.00–2.50) and ICC (OR: 2.00 95%CI:1.14–3.44) [16]. The difference in findings may be explained in part by the difference in comparison groups and outcomes in the two studies. The Tanzania study distinguished between the presence of CIN vs. no CIN and ICC vs no CIN, whereas, in the current study, the primary comparison was CIN2+ vs CIN1. Furthermore, the most advanced outcome in our study was CIN3 compared to ICC in the previous study. Mechanistically, aberrant methylation in some CpGs in the *IGF2/H19* imprinted domain may influence loss of imprinting and the exclusive use of *IGF2* promoter 1, inducing *IGF2* overexpression as previously shown [31]. The Tanzania study also showed a statistically significant association between HR-HPV infection and IGF2 DMR methylation (β = −8.55, *p* < .0001), supporting the hypothesis that aberrant DNA methylation may mediate the association between HR-HPV infection and the risk of high grade CIN and ICC. This association was not statistically significant in our study (β = 2.49, *p* = .07). Earlier analyses in this cohort, reported a negative association between aberrant DNA methylation of *IGF2AS* DMR and the regression of low-grade cervical lesions (HR = 0.41 95% CI = 0.23–0.76) [17]. We found no statistically significant association with high-grade lesions in our analyses.

A limitation of this study is its cross-sectional nature which limits the ability to infer methylation in DMRs as important factors in disease progression. However, identifying methylation markers associated with CIN2+ is an important step to allow for longitudinal evaluation of these markers in CIN progression. Secondly, we had few cases of CIN3+ and no cases of ICC in this study, which could have affected our effect estimates. In prior studies, we reported statistically significant associations between DMR methylation and ICC, and corresponding significant associations between DMR methylation and HR-HPV infections [15, 16]. Although we had CIN2+ cases (*n* = 52 CIN2 and 20 CIN3 cases) that allowed us to analyze by grade-specific CIN, research shows that approximately 40% of undiagnosed CIN2 cases will regress over time [32, 33]. Furthermore, the grading of cervical lesions, particularly CIN1 and CIN2 could be affected by discordant grading among pathologists [34]. P16 staining has shown better concordance in diagnosis [35]. However, in our study, 47% of the CIN2+ cases were stained. Thus, we cannot rule out some misclassification in grading of cervical lesions. Lastly, we were limited in statistical power to examine DMR methylation in relation to grade and ethnic-specific CIN2+, after accounting for the effect of HPV infection. Cytosine methylation at imprinted genes is normally a stable modification in human tissue samples, and therefore DMR methylation status could potentially be used as a marker to identify high-grade lesions that are likely to progress to ICC.

In summary, aberrant DNA methylation at the *PEG3* DMR may be associated with CIN2+ in HR-HPV positive cases. A study with sufficient cases of CIN3+ could confirm whether DNA methylation at this DMR represents susceptibility loci that could be exploited to identify CIN2+ cases that are likely to progress.

## Conclusions

DNA methylation analysis is a promising risk stratification strategy to distinguish HR-HPV positive women with clinically relevant cervical lesions from those with non-progressive infections. Our findings suggest that *PEG3* methylation may be associated with advanced cervical CIN lesions. Further investigations are warranted to determine its efficacy as a biomarker for cervical cancer screening.

## Supplementary Information

**Additional file 1: Supplementary Table 1.** Primers used for Sequenom analysis.

**Additional file 2: Supplementary Table 2.** Median DMR methylation levels expressed as percentages by cervical intraepithelial neoplasia (CIN) status.

**Additional file 3: Supplementary Table 3.** Crude odds ratios and receiver operator characteristic (ROC) curves for the association between the different DMRs and CIN status.

**Additional file 4: Supplementary Table 4.** Odds ratios (OR) and 95% confidence intervals (CI) for association between DMR methylation markers and CIN2+ cases with HR-HPV infection and p16 positive.

## Data Availability

The datasets analyzed during the current study available from the corresponding author on reasonable request.

## Abbreviations

AUC: Area under the curve
CI: Confidence intervals
CIN: Cervical intraepithelial neoplasia
CINCS: Cervical Intraepithelial Neoplasia Cohort Study
DMR: Differentially methylated region
HPV: Human papillomavirus
HR-HPV: High-risk human papillomavirus
ICC: Invasive cervical cancer
IQR: Interquartile range
LSIL: Low grade squamous intraepithelial lesion
LR-HPV: Low-risk human papillomavirus
OR: Odds ratio
ROC: Receiver operating characteristic
SE: Standard error
