# Supplementary material for: Association between PEG3 DNA methylation and high-grade cervical intraepithelial neoplasia
Source: Infect Agent Cancer. 2021 Jun 13;16:42. doi: 10.1186/s13027-021-00382-3 (PMC8201933; doi:10.1186/s13027-021-00382-3)
Supplement: Supplementary file 1 — Additional file 1: Supplementary Table 1. Primers used for Sequenom analysis. [file 13027_2021_382_MOESM1_ESM.docx]

**Supplementary Table 1:** Primers used for Sequenom analysis

| **Gene** | **Primer Seq.** | **Chromosome** | **Start** | **End** |
| --- | --- | --- | --- | --- |
| *H19-IGF2* | aggaagagagTATTTTGAGGTTTTGGGGGATATTA | 11 | 2130112 | 2130388 |
|  | cagtaatacgactcactatagggagaaggctCTCCCTCAACAAAAACTAACAAATC |  |  |  |
| *MESTIT1, MEST* | aggaagagagGGGTTTAGAGGTATAAGAAAGAGGG | 7 | 130130648 | 130131063 |
|  | cagtaatacgactcactatagggagaaggctTTTCTAAAAACAACCAAACCCCTAC |  |  |  |
| *Kv DMR* | aggaagagagTTTGGTAGGATTTTGTTGAGGAGTTTT | 11 | 2721161 | 2721464 |
|  | cagtaatacgactcactatagggagaaggctCTCACACCCAACCAATACCTCATAC |  |  |  |
| *MEG3* | aggaagagagTTGTGATAAGGTTAGTGAGGGGTTA | 14 | 101293947 | 101294390 |
|  | cagtaatacgactcactatagggagaaggctCCAACCAAAACCCACCTATAACTAC |  |  |  |
| *HYMAI, ZAC* | aggaagagagGAAAAAGTTTGTTTTAAGTAATAATGGGAT | 6 | 144328445 | 144328885 |
|  | cagtaatacgactcactatagggagaaggctAAAAAACCAAAACCTCAATAAAACC |  |  |  |
| *PEG10* | aggaagagagAGGTGTGGGATTTTATTTTTTTTGT | 7 | 94285845 | 94286061 |
|  | cagtaatacgactcactatagggagaaggctCAAACCTTTAAAACTTAATTTCCCC |  |  |  |
| *PEG3* | aggaagagagTATTGGGTGTTATTTTTTATGAGGG | 19 | 57350715 | 57351051 |
|  | cagtaatacgactcactatagggagaaggctTCTACTACCAACCAACCAAAACAAC |  |  |  |
