# Supplementary material for: Association between PEG3 DNA methylation and high-grade cervical intraepithelial neoplasia
Source: Infect Agent Cancer. 2021 Jun 13;16:42. doi: 10.1186/s13027-021-00382-3 (PMC8201933; doi:10.1186/s13027-021-00382-3)
Supplement: Supplementary file 2 — Additional file 2: Supplementary Table 2. Median DMR methylation levels expressed as percentages by cervical intraepithelial neoplasia (CIN) status. [file 13027_2021_382_MOESM2_ESM.docx]

**Supplementary Table 2.** Median DMR methylation levels expressed as percentages by cervical intraepithelial neoplasia (CIN) status

| DMR | Median (IQR) | |  | Median (IQR) | |
| --- | --- | --- | --- | --- | --- |
|  | CIN2+ | ≤CIN1 |  | CIN3+ | ≤CIN2 |
| *PEG3* | 29.0 (14.9) | 27.5 (11.0) |  | 26.0 (12.7) | 27.7 (12.1) |
| *PLAGLI/HYMAI* | 37.2 (11.3) | 38.0 (10.3) |  | 37.7 (7.0) | 38.0 (10.5) |
| *Kv DMR* | 31.0 (7.1) | 31.6 (6.1) |  | 30.2 (7.6) | 31.4 (6.1) |
| *IGF2/H19* | 67.8 (6.5) | 68.5 (6.3) |  | 67.8 (10.3) | 68.5 (6.3) |
| *IGF2AS* | 39.1 (14.8) | 37.3 (20.3) |  | 40.5 (9.7) | 37.7 (20.0) |
| *MESTIT1/MEST* | 34.1 (10.2) | 33.4 (7.7) |  | 30.7 (13.7) | 33.5 (7.7) |
| *PEG10* | 34.69 (8.5) | 34.4 (6.5) |  | 34.1 (7.0) | 34.6 (6.9) |
| *MEG3* | 48.4 (12.3) | 46.9 (16.7) |  | 53.6 (15.6) | 46.9 (15.9) |

Abbreviations: DMR, differentially methylated regions; IQR, interquartile range; CIN, cervical

intraepithelial neoplasia

Mann-Whitney test *p*-value >.05
