# Supplementary material for: Association between PEG3 DNA methylation and high-grade cervical intraepithelial neoplasia
Source: Infect Agent Cancer. 2021 Jun 13;16:42. doi: 10.1186/s13027-021-00382-3 (PMC8201933; doi:10.1186/s13027-021-00382-3)
Supplement: Supplementary file 3 — Additional file 3: Supplementary Table 3. Crude odds ratios and receiver operator characteristic (ROC) curves for the association between the different DMRs and CIN status. [file 13027_2021_382_MOESM3_ESM.docx]

**Supplementary Table 3.** Crude odds ratios and receiver operator characteristic (ROC) curves for the association between the different DMRs and CIN status

| **DMR** | **CIN2+ vs ≤CIN1** | |  | **CIN3+ vs ≤ CIN2** | |
| --- | --- | --- | --- | --- | --- |
|  | OR (95% CI) | ROC (95%CI) |  | OR (95%CI) | ROC (95%CI) |
| *PEG3* | 1.14 (0.86-1.52) | 0.53 (0.45-0.62) |  | 0.76 (0.44-1.32) | 0.59 (0.45-0.73) |
| *PLAGLI/HYMAI* | 0.99 (0.73-1.34) | 0.50 (0.42- 0.58) |  | 0.83 (0.45-1.54) | 0.54 (0.42- 0.67) |
| *Kv DMR* | 1.16 (0.78-1.72) | 0.52 (0.45-0.60) |  | 0.83 (0.37-1.89) | 0.54 (0.40-0.67) |
| *IGF2/H19* | 1.14 (0.75-1.72) | 0.51 (0.44-0.59) |  | 0.86 (0.44-1.70) | 0.53 (0.39-0.67) |
| *IGF2AS* | 1.10 (0.91-1.34) | 0.54 (0.46-0.62) |  | 1.13 (0.80-1.61) | 0.57(0.42-0.72) |
| *MESTIT1/MEST* | 0.98 (0.68-1.40) | 0.50 (0.42-0.57) |  | 0.87 (0.45-1.68) | 0.53 (0.37-0.69) |
| *PEG10* | 1.20 (0.78-1.83) | 0.51 (0.44-0.58) |  | 1.12 (0.50-2.49) | 0.51 (0.38-0.64) |
| *MEG3* | 1.01 (0.81-1.27) | 0.51 (0.45-0.58) |  | 1.39 (0.95-2.03) | **0.62 (0.51-0.73)** |

Abbreviations: DMR, differentially methylated regions; CIN, cervical intraepithelial neoplasia; OR, odds ratio; CI, confidence interval; ROC, receiver operator characteristic curve.
