# Supplementary material for: Association between PEG3 DNA methylation and high-grade cervical intraepithelial neoplasia
Source: Infect Agent Cancer. 2021 Jun 13;16:42. doi: 10.1186/s13027-021-00382-3 (PMC8201933; doi:10.1186/s13027-021-00382-3)
Supplement: Supplementary file 4 — Additional file 4: Supplementary Table 4. Odds ratios (OR) and 95% confidence intervals (CI) for association between DMR methylation markers and CIN2+ cases with HR-HPV infection and p16 positive. [file 13027_2021_382_MOESM4_ESM.docx]

**Supplementary Table 4**. Odds ratios (OR) and 95% confidence intervals (CI) for association between DMR methylation markers and CIN2+ cases with HR-HPV infection and p16 positive

| **DMR** | **Odds ratios (95% CI)** | | |
| --- | --- | --- | --- |
|  | **Overall** | **Black women** | **White women** |
| *PEG3* | **1.56 (1.03-2.36)** | 1.67 (0.84-3.34) | 1.51 (0.81-2.82) |
| *PLAGL1/HYMAI* | 1.03 (0.65-1.64) | 1.04 (0.47-2.31) | 1.02 (0.55-1.88) |
| *Kv DMR* | 1.31 (0.78-2.21) | 1.44 (0.72-2.87) | 1.53 (0.58-3.99) |
| *IGF2/H19* | 1.62 (0.82-3.20) | 0.89 (0.33-2.44) | 2.18 (0.91-5.22) |
| *IGF2AS* | 1.12 (0.85-1.49) | 0.87 (0.58-1.31) | 1.17 (0.80-1.71) |
| *MESTIT1/MEST* | 0.89 (0.50-1.58) | 0.53 (0.19-1.47) | 0.86 (0.43-1.70) |
| *PEG10* | 1.55 (0.77-3.12) | 2.43 (0.65-9.06) | 1.26 (0.54-2.95) |
| *MEG3* | 0.88 (0.61-1.27) | 1.16 (0.65-2.07) | 0.70 (0.42-1.19) |

ORs adjusted for age, race, smoking and parity

Abbreviations: DMR, differentially methylated regions; OR, odds ratio; CI, confidence intervals, HR HPV, high-risk human papillomavirus.
